# Dissemination of *Orientia tsutsugamushi*, a Causative Agent of Scrub Typhus, and Immunological Responses in the Humanized DRAGA Mouse

**DOI:** 10.3389/fimmu.2018.00816

**Published:** 2018-04-30

**Authors:** Le Jiang, Erin K. Morris, Rodrigo Aguilera-Olvera, Zhiwen Zhang, Teik-Chye Chan, Soumya Shashikumar, Chien-Chung Chao, Sofia A. Casares, Wei-Mei Ching

**Affiliations:** ^1^Viral and Rickettsial Diseases Department, Naval Medical Research Center, Silver Spring, MD, United States; ^2^Veterinary Services Program, Department of Pathology Services, Walter Reed Army Institute of Research, Silver Spring, MD, United States; ^3^US Military Malaria Vaccine Program, Naval Medical Research Center, Walter Reed Army Institute of Research, Silver Spring, MD, United States; ^4^Uniformed Services University of the Health Sciences, Bethesda, MD, United States

**Keywords:** scrub typhus, *Orientia*, mouse model, humanized mice, footpad inoculation

## Abstract

Scrub typhus is caused by *Orientia tsutsugamushi*, an obligated intracellular bacterium that affects over one million people per year. Several mouse models have been used to study its pathogenesis, disease immunology, and for testing vaccine candidates. However, due to the intrinsic differences between the immune systems in mouse and human, these mouse models could not faithfully mimic the pathology and immunological responses developed by human patients, limiting their value in both basic and translational studies. In this study, we have tested for the first time, a new humanized mouse model through footpad inoculation of *O. tsutsugamushi* in DRAGA (HLA-A2.HLA-DR4.Rag1KO.IL2RγcKO.NOD) mice with their human immune system reconstituted by infusion of HLA-matched human hematopoietic stem cells from umbilical cord blood. Upon infection, *Orientia* disseminated into various organs of DRAGA mice resulted in lethality in a dose-dependent manner, while all C3H/HeJ mice infected by the same route survived. Tissue-specific lesions associated with inflammation and/or necroses were observed in multiple organs of infected DRAGA mice. Consistent with the intracellular nature of *Orientia*, strong Th1, but subdued Th2 responses were elicited as reflected by the human cytokine profiles in sera from infected mice. Interestingly, the percentage of both activated and regulatory (CD4^+^FOXP3^+^) human T cells were elevated in spleen tissues of infected mice. After immunization with irradiated whole cell *Orientia*, humanized DRAGA mice showed a significant activation of human T cells as evidenced by increased number of human CD4^+^ and CD8^+^ T cells. Specific human IgM and IgG antibodies were developed after repetitive immunization. The humanized DRAGA mouse model represents a new pre-clinical model for studying *Orientia*-human interactions and also for testing vaccines and novel therapeutics for scrub typhus.

## Introduction

Scrub typhus is an infectious disease, affecting over one million people per year and putting over a billion people at risk in its endemic areas (1). It is also of military significance and historically has been a leading cause of morbidity and mortality during warfare in the Asia-Pacific region. Patients with scrub typhus often display symptoms, including fever, eschar, headache, rash, pneumonitis, and lymphadenopathy. If diagnosed early, it can be effectively treated with antibiotics, such as doxycycline, delayed treatment; however, can be lethal (2). Recent outbreaks of scrub typhus in endemic regions and emergence of this disease in non-traditional areas, such as Middle East (3), South America (4), and Africa (5) have emphasized the importance for early diagnosis, disease control, and treatment. Furthermore, no vaccine is currently available, mainly due to strain variations which lead to antigenic heterogeneity and short-term immunity (6). Insight into the pathogenesis and immunological responses upon *Orientia* infection is paramount not only in understanding its disease progression, but also for developing preventive strategies and novel therapeutics.

Scrub typhus is caused by *Orientia*, an obligate intracellular Gram-negative bacterium transmitted to human by chigger, the larval stage of *Leptotrombidium* mite (7). Due to the short length of their mouth pieces, chiggers can only reach the epidermis part of the skin (8), where *Orientia* will enter the host during feeding. In certain percentage of patients, this will elicit strong local immunological reactions leading to the formation of eschars at the bite site. Examination of the composite structure of eschar has suggested that dendritic cells and monocytes/macrophages might be the major early host cells to encounter and harbor *Orientia* (9). The intracellular bacteria appear to possess the ability to escape from host defense mechanisms and proliferate in these antigen presenting cells including macrophages (10). In a matter of days, they can disseminate to distant major organs presumably *via* both lymphatic and hematologic circulatory systems.

Numerous animal models have been developed to study scrub typhus, including mice, rats, rabbits, monkeys, etc. (11). Monkeys are probably the best model, but they are very expensive and require specialized facilities (12). Mice are most frequently used due to their low cost and ease to handle. Mouse models have been very instrumental in previous studies looking into pathogenesis (13), vaccine tests (14) and more recently, into dissecting functions and mechanisms of specific immunological events (15). However, mouse models could not faithfully mimic the immunological reactions developed by human patients. This is probably due to the fundamental differences between the two species, especially in terms of their immune system makeup (16). These differences make it difficult to apply knowledge gained with mouse model to humans. Utilizing humanized mice could probably bridge this gap and hold the promise of providing more relevant models for immunological studies and vaccine development (17).

Earlier generations of human immune system humanized mice showed poor ability to support reconstitution and development of functional human T cells or B cells that are able to secrete IgG (17). With the introduction of HLA transgenes in the Rag1KO.IL2RγcKO.NOD (NRG) background, the DRAG mice (HLA-DR4.Rag1KO.IL2RγcKO.NOD), and DRAGA mice (HLA-A2.HLA-DR4.Rag1KO.IL2RγcKO.NOD) infused with HLA-matched human hematopoietic stem cells (HSC) have been shown to repopulate the mouse thymus and to reconstitute functional human T cells that support human B cell immunoglobulin class switching and secretion of human IgG (18–24). These mice have been used successfully for supporting infection with human pathogens, such as *Plasmodium falciparum* (malaria), HIV, Zika, and influenza A virus, and for analyzing human immune responses upon infection or vaccination (20–25). In this study, we successfully established a lethal challenge mouse model in humanized DRAGA mice using footpad inoculation of *Orientia tsutsugamushi* (*O. tsutsugamushi*) (Karp strain). We showed live *Orientia* dissemination into major organs of DRAGA mice, which caused pathological changes in various tissues. More importantly, *Orientia* infection induced human immune responses, including T cell activation, cytokine secretion, and specific antibody development.

## Materials and Methods

### Generation of Humanized DRAGA Mice and Ethics Statement

DRAGA mice express HLA-A2.1 and HLA-DR0401 molecules on a NRG background and they have been previously described (19, 22, 24). HLA-A2.1/HLA-DR0401 positive umbilical cord blood was obtained from the NY Blood Center, Long Island City. Four- to six-week-old DRAGA mice were irradiated (350 rads) and injected intravenously with CD3 T cell-depleted cord blood cells (EasySep Human CD3 Positive Selection Kit, Stem Cell Technologies, #18051) containing approximately 10^5^ human hematopoietic stem cells (HSCs) (CD34^+^) as measured by fluorescence-activated cell shorting using human CD34 antibodies (clone #563, BD Biosciences). The procedures for assessing human immune cell reconstitution in peripheral blood have been previously described (18, 19). DRAGA mice were used for 4 months after infusion of human HSCs. All animal procedures reported herein were conducted under IACUC protocols approved by WRAIR/NMRC in compliance with the Animal Welfare Act and in accordance with the principles set forth in the “Guide for the Care and Use of Laboratory Animals,” Institute of Laboratory Animals Resources, National Research Council, National Academy Press, 2011.

### *Orientia* Inoculum Preparation and Footpad Inoculation

All procedures involved using live *Orientia* was performed in biosafety level 3 (BSL-3) laboratories. *Orientia* (Karp strain) inoculum that was purified previously (14) was injected into peritoneal cavity of C3H/HeJ mice. Mice were euthanized 7 to 10 days post inoculation. Liver and spleen tissues were homogenized in SYN1 buffer (0.22 M sucrose, 3.6 mM KH_2_PO_4_, 8.6 mM Na_2_HPO_4_, and 4.9 mM glutamic acid) at 1:20 ratio (v/v) and then aliquoted and stored in −80°C freezer until use. Serial dilution of the inoculum were used for intraperitoneal (i.p.) inoculation in CD-1 mice to calculate mLD^50^ (26) and also quantified by quantitative PCR (qPCR). Inoculation of humanized DRAGA mice was performed by injecting 30 µL inoculum into each footpad (total 60 µL per mouse). Homogenized liver and spleen tissues from non-infected C3H/HeJ mice were injected as controls.

### Quantification of *Orientia*

DNA from bone marrow and major tissues were extracted using blood/tissue DNA kit (Qiagen) and qPCR targeting 47 kDa gene of *Orientia* were performed on a 7500 Fast Real-Time PCR System (27). Serial dilutions of plasmid containing the amplification fragment sequence (28) was used to generate standard curves for absolute copy number quantification.

### Cell Culture and Bacterial Infection

L929 mouse fibroblast cells originally from ATCC were maintained in DMEM media containing 10% FBS, 100 U/mL penicillin, and 100 µg/mL streptomycin. Cells were cultured in incubator at 37°C with 5% CO_2_. Antibiotics were removed from cell culture 24 h prior to infection. Two microliters of inoculum prepared from humanized DRAGA lung tissues were added to a T25 flask containing L929 cells and rocked for 1 h at room temperature and used for immunofluorescent staining on day 7 and day 14.

### Immunofluorescent and Immunohistochemistry

Cultured L929 cells or frozen lung and liver sections on glass slides were fixed with 4% paraformaldehyde for 20 min and permeabilized with 0.1% Triton for 5 min at room temperature. They were then blocked in 1% BSA in PBS for 45 min before incubation with scrub typhus patient sera as primary antibody (pooled on day 11 post onset of fever and diluted at 1:1,000) at room temperature for 1 h. The slides were then washed three times (5 min each) in PBS before incubating with a goat anti-human IgG secondary antibody conjugated with Alexa Fluor 568 (Thermo Fisher Scientific). The slides were then washed three times (5 min each) in PBS before being examined under a fluorescent microscope. For immunohistochemistry, animal tissues were fixed in formalin overnight and replaced with 70% ethanol the next day. Paraffin-embedded tissue sections from humanized DRAGA mice post challenge were cut into 5 μm sections and stained with hematoxylin and eosin (H&E).

### Immunization

*Orientia* (Karp stain) inoculum was irradiated at 200 krads for inactivation. Inoculum prepared from uninfected mouse was irradiated as well and used as the control. A total of four immunizations were performed with 2-week intervals. For each immunization, 1 × 10^7^ irradiated bacteria were injected intraperitoneally into each humanized DRAGA mouse.

### Antibody Detection and Characterization

Blood from each mouse was collected *via* tail vein. Enzyme-linked immunosorbent assay (ELISA) was used to monitor the development of IgM and IgG specific to 56 kDa recombinant protein (14). Positive sera were confirmed by immunofluorescence assay on glass slides spotted with whole cell antigen of *Orientia*.

### Cytokine Profiling

Cytokines and chemokines from humanized DRAGA mouse sera were profiled using Bio-Plex Pro human cytokine 17-plex Assay (Bio-Rad) in a MAGPIX system (Luminex). To ensure mouse cytokines were not interfering with the results, sera from CD-1 mice with or without *Orientia* infection were included as negative controls.

### Flow Cytometry

Blood (50 µL) from tail vein was collected using heparin-coated capillary tubes, spun down, and erythrocytes were lysed with ACK buffer for 5 min in ice followed by a washed with 1% BSA in PBS. Splenocytes were isolated as previously described (19). Cells were blocked with anti-mouse Fc block (BD Biosciences) and surface stained with antibodies against human CD3 (#HIT3a), CD4 (#SK3), CD8 (#RPA-T8), CD69 (#L78), CD62L (#DREG-56), and CCR7/CD197 (#150503) from BD Biosciences as described (18–20). To evaluate the frequency of human CD4^+^FOXP3^+^ regulatory T cells in spleens of DRAGA mice, cells were first surface stained with human CD3, CD4 antibodies, and then intracellularly stained with a antibody against human FOXP3 (#236A/E7, Thermo Fisher Scientific) following the manufacturer’s instructions. Cells were analyzed in the gated mononuclear FSC/SSC as described previously (19).

### Statistical Analysis

Comparison between two groups of data was performed using Welch’s *t*-test in Graphpad Prism 7 software. A *p* value less than 0.05 was considered significant.

## Results

### Footpad Inoculation of *O. tsutsugamushi* Causes Lethality in Humanized DRAGA Mice, But Not in C3H/HeJ Mice

**Figure 1:**
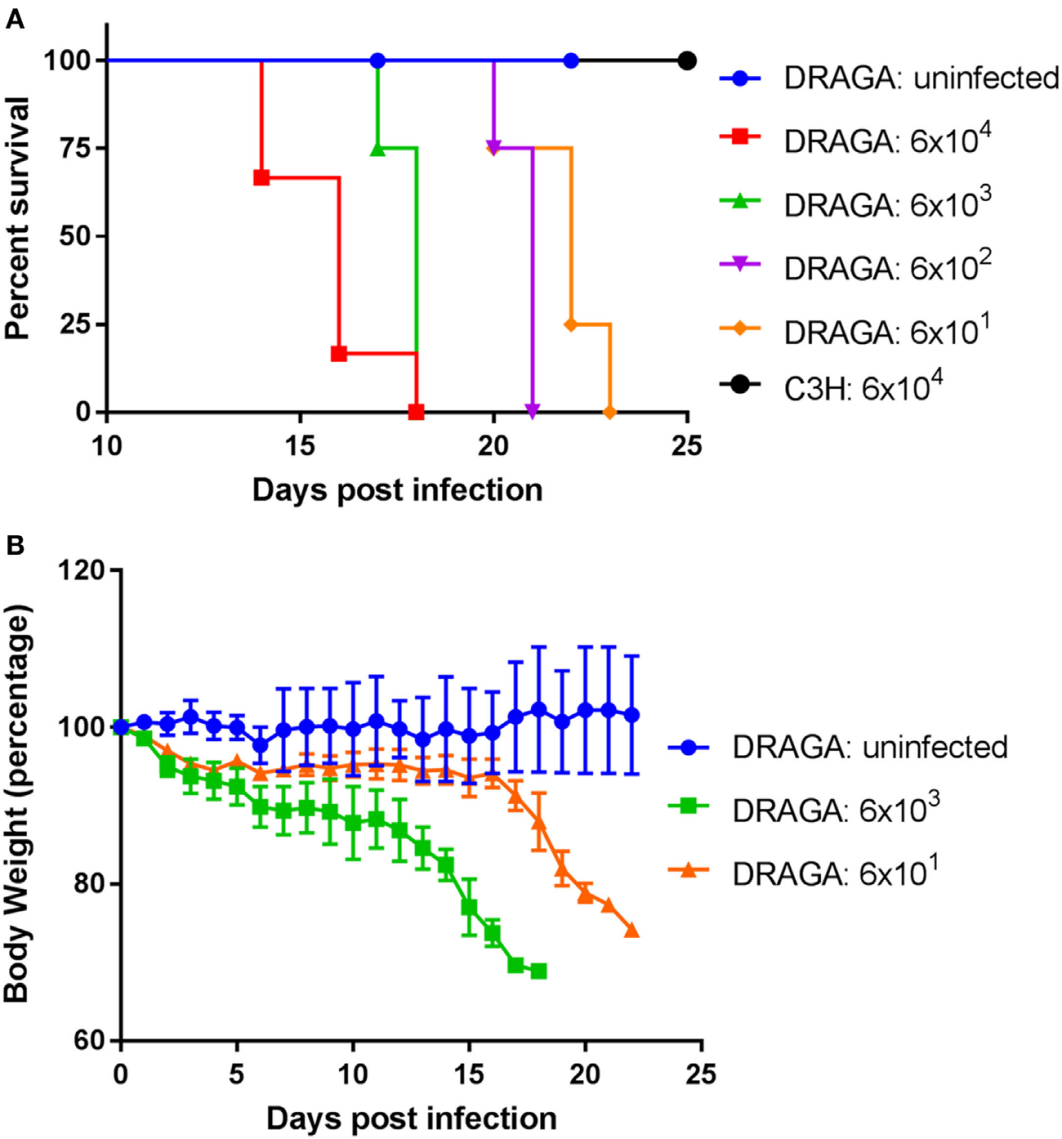
A lethal model for scrub typhus using humanized DRAGA mice. **(A)** Survival curve of humanized DRAGA and C3H mice inoculated *via* footpad with vehicle control or *Orientia tsutsugamushi* (Karp) of various dosages (mLD^50^): 6 × 10^4^ (DRAGA, *n* = 6; C3H, *n* = 4), 6 × 10^3^ (*n* = 4), 6 × 10^2^ (*n* = 4), and 6 × 10^1^ (*n* = 4). **(B)** The body weight was monitored daily after infection with vehicle control or *Orientia* inoculum at mLD^50^ of 6 × 10^3^ and 6 × 10^1^.

Footpad inoculation has been used in recent mouse models to study inflammatory responses induced by *O. tsutsugamushi* (29). It is considered as a route that combines intradermal and subcutaneous inoculation (30), which mimics the natural way of infection *via* chigger bites. Humanized DRAGA mice were inoculated with inoculum (liver/spleen homogenate) containing various amount of *Orientia* ranging from 6 × 10^1^ to 6 × 10^4^ mLD^50^ *via* footpad. For the control group, liver/spleen homogenate that does not contain any bacteria were injected. As illustrated in Figure 1A, mice challenged with the highest dose of *Orientia* (6 × 10^4^) group began to show signs of illness such as ruffled fur on day 11 infection and succumbed to infection starting from day 14 post infection. By day 18, all six mice in this group died. However, when inoculated into inbred C3H/HeJ mice, the same high dose did not cause any lethality. This is consistent to what has been reported in inbred BALB/c mice upon footpad inoculation, where the infected BALB/c mice did not die due to infection (29). Lower *Orientia* challenge doses in humanized DRAGA mice substantially delayed the appearance of sickness and eventual lethality (Figure 1A). Over the course of the infection, the body weights of DRAGA mice decreased gradually and this reduction accelerated during the last 4–5 days before death (Figure 1B). These data indicated that *Orientia* caused dose-dependent lethality in footpad-inoculated humanized DRAGA mice, but not in C3H/HeJ or in BALB/c mice.

### *Orientia* Disseminates Into Major Organs of Humanized DRAGA Mice

**Figure 2:**
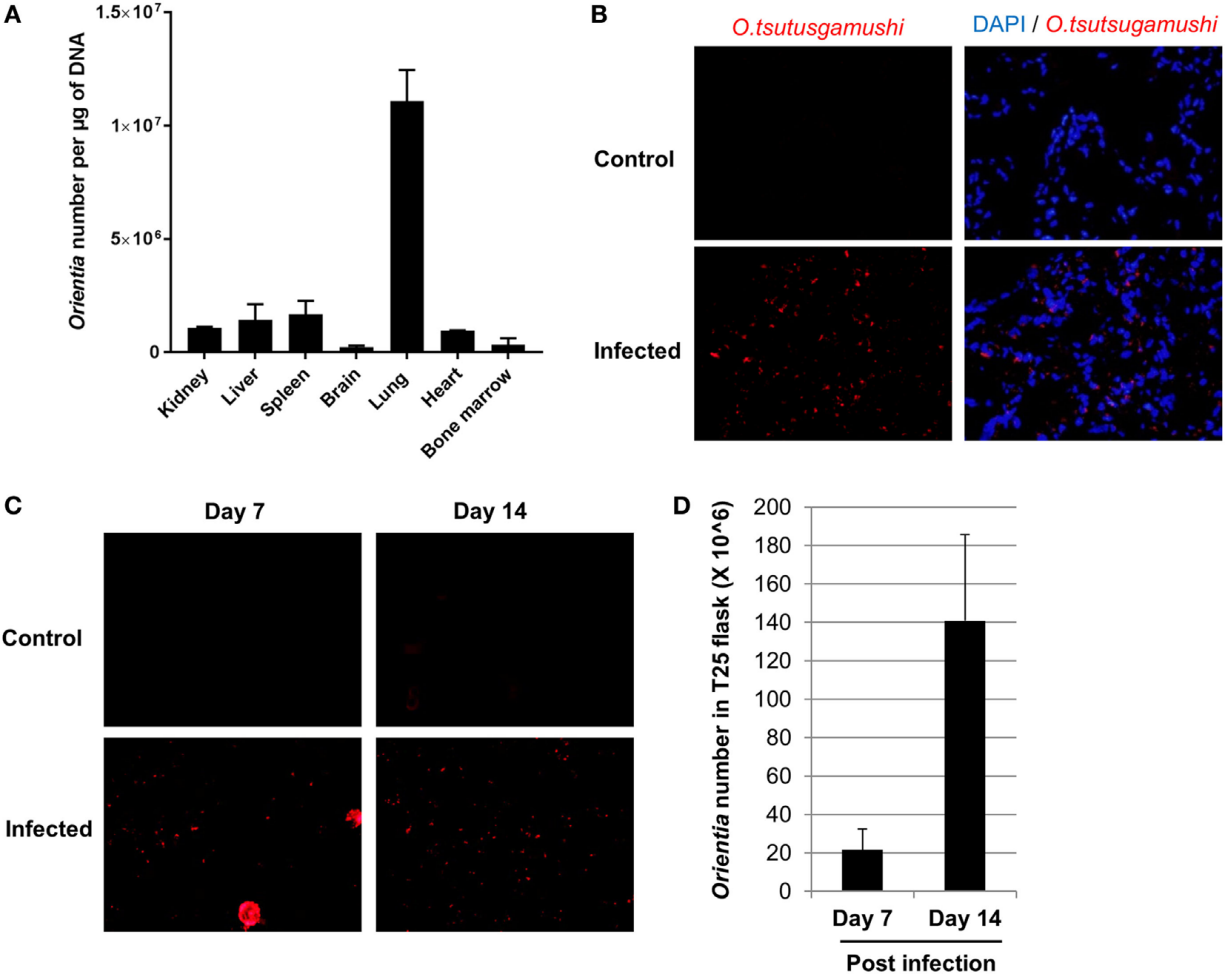
Live *Orientia tsutsugamushi* disseminates into organs of infected humanized DRAGA mice. **(A)** DNA was extracted from various organs of humanized DRAGA mice 2 weeks post infection at 6 × 10^4^ mLD^50^ and *O. tsutsugamushi* was quantified by quantitative PCR (qPCR). **(B)** Immunofluorescence staining showing *O. tsutsugamushi* (red) in lung tissue frozen sections from humanized DRAGA mice 3 weeks post infection at 6 × 10^2^ mLD^50^ (cell nuclei were stained blue with DAPI, magnification, 400×). **(C)** Tissue homogenates were prepared from lung tissue and used to inoculate L929 cells. Immunofluorescence staining was performed to detect the presence of *O. tsutsugamushi* (magnification, 400×) at day 7 and day 14 post inoculation. **(D)** qPCR was used to quantify number of *Orientia* in L929 cells collected in **(C)**.

In order to investigate tissue tropism of *Orientia*, we harvested tissues when humanized DRAGA mice became severely sick after infection. DNA was extracted and the number of *Orientia* was quantified by qPCR. As illustrated in Figure 2A, lung was found to contain the most number of bacteria, followed by spleen, liver, kidney, and heart. Brain and bone marrow had the least number of bacteria among the tissues examined. Furthermore, immunofluorescent staining of *Orientia* in frozen tissue sections showed extensive proliferation of bacteria in about 50% of cells in the lung (Figure 2B) and to a lesser extent in liver cells (Figure S1 in Supplementary Material). To further test the infectivity of *Orientia* isolated from infected DRAGA mice, tissue homogenate from lung tissue was prepared and used to inoculate L929 cells, a common cell line for culturing *Orientia*. As illustrated in Figures 2C,D, L929 cells were infected with *Orientia* as indicated by specific staining (Figure 2C) and proliferation of *Orientia* in these cells were quantified by qPCR (Figure 2D). These results clearly indicated that humanized DRAGA mice sustain infection with *Orientia*.

### Pathological Changes in Humanized DRAGA Mice due to *Orientia* Infection

**Figure 3:**
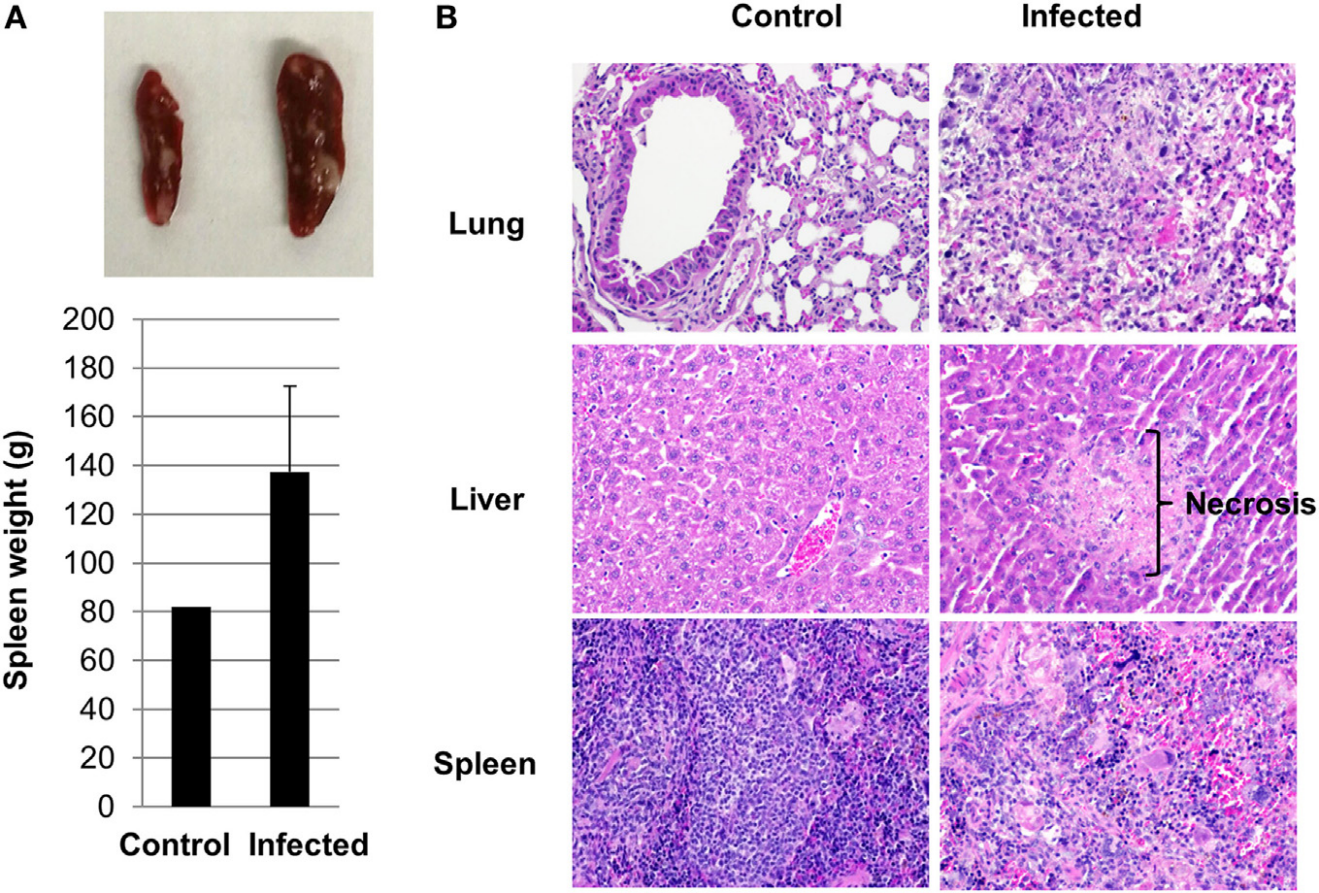
Pathological changes in infected humanized DRAGA mice. **(A)** A representative photo of splenomegaly and average spleen organ weight from control and infected humanized DRAGA mice when they were severely sick. **(B)** Hematoxylin and eosin staining reveals pathological changes in organs of humanized DRAGA mice infected with *Orientia tsutsugamushi* (magnification, 200×).

Splenomegaly was apparent in infected mice when they became severely sick. The weight of the spleens ranged from 92 to 196 g in the infected group compared to around 80 g in the controls (Figure 3A). Histologic findings on tissue sections stained with H&E revealed inflammation and necrosis in the lung, liver, and spleen. Areas of pyogranulomatous and necrotizing splenitis were multifocal to coalescing (Figure 3B). There was mild to moderate red pulp necrosis, characterized by cellular debris, neutrophils, and multinucleated giant cells, admixed with increased extramedullary hematopoiesis (EMH) that crowded out normal lymphocytes. The EMH was characterized by abundant hematopoietic precursor cells, megakaryocytes, and intracellular hemosiderin due to increased erythrocyte breakdown (Figures S2A,B in Supplementary Material). Multifocal, miliary, and random necrosis in hepatocytes was clearly identifiable in infected liver associated with fibrinosuppurative and lymphohistiocytic inflammation. Hepatocytes within necrotic foci frequently contained intracellular bacteria, which were confirmed with Gram stains. Infected lungs in humanized DRAGA mice displayed perivasculitis, edema, fibrin, and hemorrhage, and multifocal foci of septal necrosis (Figure 3B; Figures S2C,D in Supplementary Material). Interestingly, no histologic changes were noted in the kidney, heart, and brain, where much less amount of bacteria were present as quantified in Figure 2A.

### Strong Th1 but Subdued Th2 Human Cytokine Regulation in Humanized DRAGA Mice Upon *Orientia* Infection

**Figure 4:**
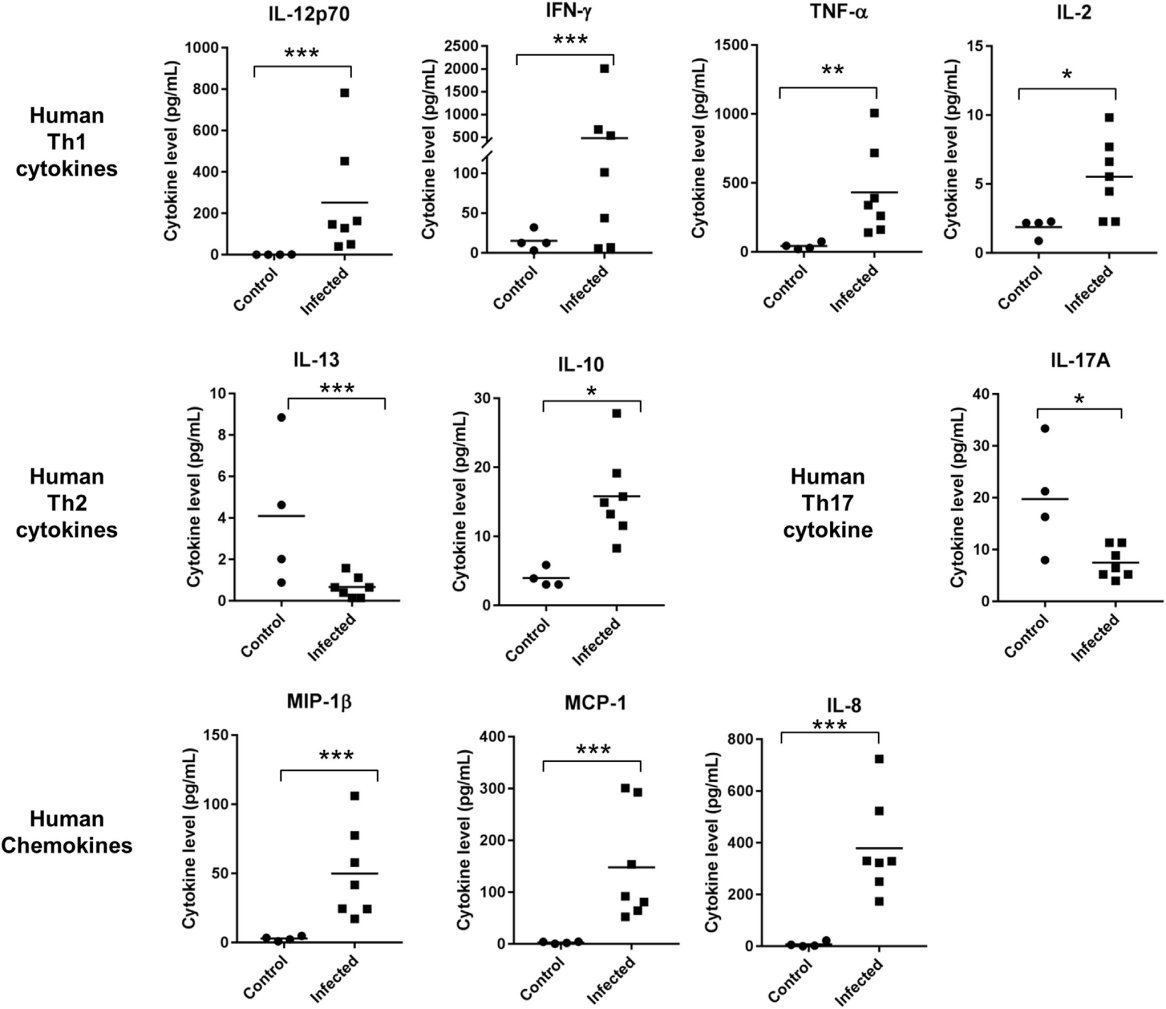
Cytokine and chemokine levels were significantly regulated in sera collected from control (*n* = 4) and infected humanized DRAGA mice (*n* = 7) (*p value < 0.05; **p value < 0.01; ***p value < 0.001).

Cytokines and chemokines are small proteins released by immune cells such as T helper cells and macrophages. They are critical in cell signaling involved in recruiting, regulating immune cells, and modulating inflammatory reactions that act upon pathogen invasions (31). Using a multiplex assay, we measured cytokine levels in sera collected from control and infected DRAGA mice. Strong Th1-cytokine responses were induced, including IL-2, IL12, IFN-γ, and TNF-α. The average levels of human IFN-γ and TNF-α were over 10-fold in the infected versus the control group. Levels of human IL-12p70 increased by 100-folds in the sera of infected mice, as compared to control (uninfected) mice. However, levels of cytokines for Th2 responses had either modest increase, such as IL-10 or were inhibited, such as IL-13 (Figure 4). Serum concentration of IL-4 were either below 0.6 pg/mL or mostly undetectable in the infected or control mice. Other cytokines or chemokines that had significant increase due to *Orientia* infection included IL-8, MIP-1β, MCP-1, IL-1β, IL-6, and G-CSF. An important player for pro-inflammatory Th17 cells, IL-17A, significantly decreased due to the infection. GM-CSF level remained unchanged (Figure 4; Figure S3 in Supplementary Material). Consistent with these cytokine profiles, the percentage of cells expressing CD69 and CD62L increased in the spleen for both CD4^+^ and CD8^+^ T cell subsets (Figure S4 in Supplementary Material). Intriguingly, the percentage of human T regulatory cells (Tregs) (CD4^+^FOXP3^+^) significantly increased in the spleen of infected mice as well (2.2 to 7.6%).

### Antigen-Specific Humoral and Cellular Responses Are Developed in Humanized DRAGA Mice Post Immunization

**Figure 5:**
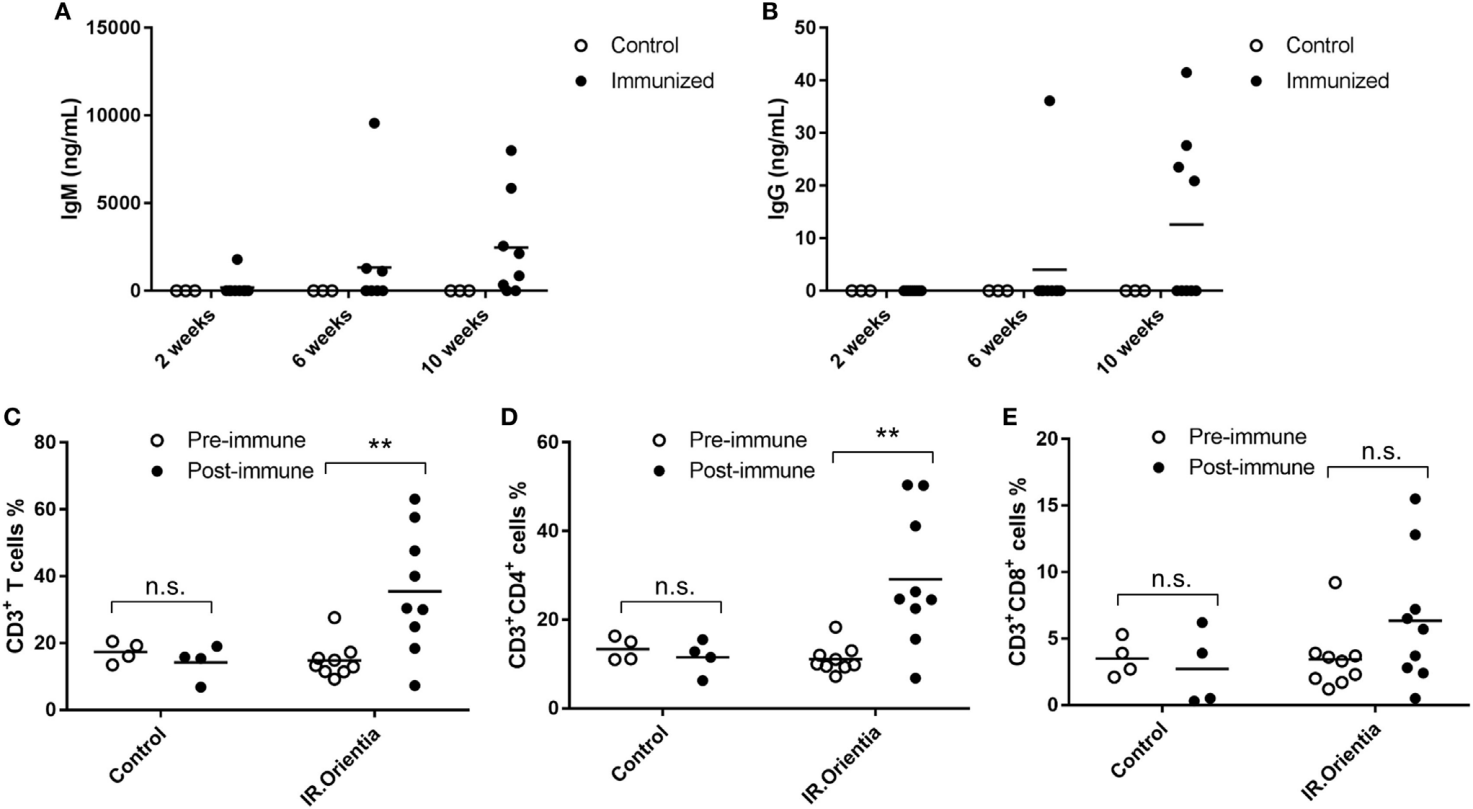
Antibody and T cell responses after immunization with irradiated whole cell *Orientia*. Concentrations for IgM **(A)** and IgG **(B)** specifically against 56 kDa protein in serum collected at 2, 6, and 10 weeks after initial immunization. **(C–E)** Percentage of CD3^+^, CD4^+^, and CD8^+^ T cells among total mononuclear cells in blood (**p value < 0.01; n.s, not significant).

Previous studies using serum or cell transfer experiments suggested that both antibody and T-cell responses were important for effective control of *Orientia* growth and provided protection in conventional mouse models by i.p. infection route (32–34). In order to evaluate these critical immune functions in humanized DRAGA mice, we immunized them with whole cell *Orientia* inactivated by irradiation (200 krads), which has been shown as effective immunogens previously (35). In inbred BALB/c mice, specific antibodies were detected 3 weeks after the initial immunization using irradiated *Orienti*a although antibody levels were much lower when compared to viable organisms (36). ELISA using a recombinant 56 kDa antigen, the dominant surface antigen for *Orientia*, showed that human IgM developed between 2 and 4 weeks after initial immunization and by 10 weeks, both human IgM and IgG were readily detectable in the sera of immunized DRAGA mice. The serum levels of human antibodies reactive to recombinant 56 kDa antigen were quantified based on a standard curve generated from a humanized monoclonal antibody against 56 kDa antigen with known concentrations (Figures 5A,B). Furthermore, significant human T cell activation was observed in the blood from immunized DRAGA mice. Percentage of human CD3^+^ T cells more than doubled 8 weeks post initial immunization and this was accompanied with a significant increase of CD4^+^ T cells (~3-fold) and moderate increase for cytotoxic CD8^+^ T cells (~2-fold), although it did not reach statistical significance (Figures 5C–E).

## Discussion

Although mouse models have been extensively used for scrub typhus research for many years, there is an urgent need for the development of an animal model that represent the pathology and immune responses of human scrub typhus (6). Given the vast differences in immune system between mouse and human species (16) and recent advancements in generating human immune system humanized mice, we aimed to develop a humanized mouse model for scrub typhus. To the best of our knowledge, this is the first of such study in the field. The fact that footpad *Orientia* inoculation leads to lethality in the humanized DRAGA mice, but not in C3H or BALB/c mice (29) very likely is due to differences of their immune systems. In this study, we have shown that humanized DRAGA mice could be infected by *Orientia via* footpad followed by dissemination into major organs. This caused severe pathological changes in liver, lung, and spleen, but not in organs with less bacterium load, such as brain, heart, and kidney. Th1-dominant human cytokines were induced dramatically in response to the inoculation. Both humoral and cellular adaptive immune responses were observed when humanized DRAGA mice were immunized by irradiated whole cell antigen.

Regulation of cytokines in patient sera has been reported for scrub typhus. Consistent with the intracellular nature of *Orientia* infection, Th1 cytokines, such as IFN-γ, TNF-α, and IL-12 were significantly upregulated while cytokines in Th2 category were much less regulated (37, 38). A very similar pattern of cytokine regulation was observed in our humanized DRAGA mice upon footpad inoculation. These included marked increase of IFN-γ, TNF-α, and IL-12 (Th1 cytokines), and moderate induction of IL-10 and suppression of IL-13 (Th2 cytokines) (Figure 4). Serum levels of IL-4, which is responsible for triggering Th2 differentiation, were mostly below the lower detection limit of our assay and thus too low to be determined. IL-10, which is considered to be a Th2 cytokine and anti-inflammatory, was moderately elevated probably due to the fact that in humans both Th1 and Th2 cells can produce IL-10 (39). Increase of IL-10 was found previously in scrub typhus patients as well (38). IL-17A, a pro-inflammatory cytokine secreted by Th17 cells was significantly inhibited in the infected DRAGA mice. Recent profiling in scrub typhus patients’ sera identified three human chemokines (MCP-1, MIP-1β, and IL-8) that were upregulated during scrub typhus infection and associated with disease severity and mortality (40). Intriguingly, all three chemokines were dramatically upregulated in our mouse model, suggestive of severe infections in our humanized DRAGA mice. Although investigations into cytokine regulation using conventional mouse models also mimicked human situations to certain extent (41), they failed to identify important regulators, such as IL-8 (as a marker of scrub typhus disease severity) which is not present in the mouse immune system (42).

Peripheral T cells in scrub typhus patient blood have been characterized recently (43), where the percentage of both CD4^+^ and CD8^+^ T cells decreased due to programmed cell death (apoptosis) during the acute phase of infection. Similar reduction of CD4^+^ and CD8^+^ T cells occurred in the spleen of humanized DRAGA mice infected with *Orientia* (Figure S4A in Supplementary Material). More interestingly, as central mediators of immune suppression, human Tregs significantly increased in the spleen upon *Orientia* infection (Figure S4C in Supplementary Material). Stimulation of CD4^+^FOXP3^+^ Tregs upon host/pathogen interaction have been reported in many infectious diseases (44), and it could have multifold impact on protecting the human host from excessive inflammation and at the same time, serving as a mechanism for pathogens to evade human immune system, which increases the risk of pathogen persistence and chronic disease. Given the severe tissue damages in humanized DRAGA mice (Figure 3; Figure S2 in Supplementary Material), increase of Tregs in these tissues might have protective effect for the host. Scrub typhus patients were documented to display significant decrease of Tregs in the peripheral blood (43) and the authors postulated that this reduction might be due to the migration of Tregs into tissues. It will be interesting to test this hypothesis in our humanized DRAGA mouse models. Functional investigations of Tregs in scrub typhus may provide further insights into its pathogenesis and immune regulation.

T cell responses have been recently shown to be critical in controlling *Orientia* growth and scrub typhus disease progression (45, 46). The marked increase of CD3^+^ T cells, along with increases in both CD4^+^ and CD8^+^ cell populations upon immunization (Figure 5), suggests that the humanized DRAGA mice might be suitable for future vaccine or mechanistic studies. In addition, tissue damage caused by necrotic cell death was seen in multiple organs of humanized DRAGA mice post infection (Figure 3B; Figure S2 in Supplementary Material). It has been shown recently by Hauptmann et al. that hepatocellular injury and subcapsular necrotic lesions were caused by CD8^+^ T cells (45). It is very likely that similar lesions observed in the liver tissues (Figure 3B) were triggered by CD8^+^ T cells as well. Given the appropriate human T cell and B cell responses after immunization (Figure 5), it will be very intriguing to test whether they will offer protection against live *Orientia* challenge.

Through the many failures of translating knowledge gained in conventional mouse models to human clinical studies (16), the difference between mouse and human (especially with regards to their immune systems) has been increasingly appreciated. For example, formation of granulomas that resemble those observed in human mycobacteriosis was only observed in humanized mice, but not in non-humanized infected controls (47). Humanized mouse models might bridge this gap (48). We are in urgent need of mouse models that can better mimic the disease pathology and immunological responses for many human diseases, including scrub typhus. This study represents a successful attempt in this effort. *Orientia* dissemination, disease pathology, cytokine regulations, and adapted immune responses as observed in humanized DRAGA mice make it a valuable tool in future basic and preclinical research for scrub typhus.

## Ethics Statement

All animal procedures reported herein were conducted under IACUC protocols approved by WRAIR/NMRC in compliance with the Animal Welfare Act and in accordance with the principles set forth in the “Guide for the Care and Use of Laboratory Animals,” Institute of Laboratory Animals Resources, National Research Council, National Academy Press, 2011.

## Author Contributions

WMC and SC conceived the study. LJ, ZZ, TCC, and SS performed the experiments. CCC, LJ, EM, SC, and WMC analyzed and interpreted data. SC and RAO provided the humanized DRAGA mice for the study. LJ wrote the manuscript with contributions from all authors.

## Conflict of Interest Statement

The authors declare that the research was conducted in the absence of any commercial or financial relationships that could be construed as a potential conflict of interest.

## References

[1] Watt G Parola P. Scrub typhus and tropical rickettsioses. Curr Opin Infect Dis (2003) 16:429–36. 10.1097/01.qco.0000092814.64370.70 14501995

[2] Taylor AJ Paris DH Newton PN. A systematic review of mortality from untreated scrub typhus (*Orientia tsutsugamushi*). PLoS Negl Trop Dis (2015) 9:e0003971. 10.1371/journal.pntd.0003971 26274584 PMC4537241

[3] Izzard L Fuller A Blacksell SD Paris DH Richards AL Aukkanit N Isolation of a novel *Orientia* species (O. chuto sp. nov.) from a patient infected in Dubai. J Clin Microbiol (2010) 48:4404–9. 10.1128/JCM.01526-10 20926708 PMC3008486

[4] Weitzel T Dittrich S López J Phuklia W Martinez-Valdebenito C Velásquez K Endemic scrub typhus in South America. N Engl J Med (2016) 375:954–61. 10.1056/NEJMoa1603657 27602667

[5] Horton KC Jiang J Maina A Dueger E Zayed A Ahmed AA Evidence of *Rickettsia* and *Orientia* infections among abattoir workers in Djibouti. Am J Trop Med Hyg (2016) 95:462–5. 10.4269/ajtmh.15-0775 27273647 PMC4973201

[6] Paris DH Shelite TR Day NP Walker DH Unresolved problems related to scrub typhus: a seriously neglected life-threatening disease. Am J Trop Med Hyg (2013) 89:301–7. 10.4269/ajtmh.13-0064 23926142 PMC3741252

[7] Traub R Wisseman CL The ecology of chigger-borne rickettsiosis (scrub typhus). J Med Entomol (1974) 11:237–303. 10.1093/jmedent/11.3.237 4212400

[8] Shatrov AB Takahashi M Noda S Misumi H Stylostome organization in feeding *Leptotrombidium larvae* (Acariformes: Trombiculidae). Exp Appl Acarol (2014) 64:33–47. 10.1007/s10493-014-9809-8 24687177

[9] Paris DH Phetsouvanh R Tanganuchitcharnchai A Jones M Jenjaroen K Vongsouvath M *Orientia* tsutsugamushi in human scrub typhus eschars shows tropism for dendritic cells and monocytes rather than endothelium. PLoS Negl Trop Dis (2012) 6:e1466. 10.1371/journal.pntd.0001466 22253938 PMC3254662

[10] Fukuhara M Fukazawa M Tamura A Nakamura T Urakami H. Survival of two *Orientia tsutsugamushi* bacterial strains that infect mouse macrophages with varying degrees of virulence. Microb Pathog (2005) 39:177–87. 10.1016/j.micpath.2005.08.004 16165341

[11] Ridgway RL Oaks SC LaBarre DD. Laboratory animal models for human scrub typhus. Lab Anim Sci (1986) 36:481–5. 3534444

[12] Robinson DM Chan TC Huxsoll DL. Clinical response of silvered leaf monkeys (*Presbytis cristatus*) to infection with strains of *Rickettsia tsutsugamushi* virulent and avirulent for mice. J Infect Dis (1976) 134:193–7. 10.1093/infdis/134.2.193 823272

[13] Shelite TR Saito TB Mendell NL Gong B Xu G Soong L Hematogenously disseminated *Orientia tsutsugamushi*-infected murine model of scrub typhus [corrected]. PLoS Negl Trop Dis (2014) 8:e2966. 10.1371/journal.pntd.0002966 25010338 PMC4091938

[14] Ni Y-S Chan T-C Chao C-C Richards AL Dasch GA Ching W-M. Protection against scrub typhus by a plasmid vaccine encoding the 56-KD outer membrane protein antigen gene. Am J Trop Med Hyg (2005) 73:936–41. 10.4269/ajtmh.2005.73.936 16282307

[15] Shelite TR Liang Y Wang H Mendell NL Trent BJ Sun J IL-33-dependent endothelial activation contributes to apoptosis and renal injury in *Orientia tsutsugamushi*-infected mice. PLoS Negl Trop Dis (2016) 10:e0004467. 10.1371/journal.pntd.0004467 26943125 PMC4778942

[16] Mestas J Hughes CCW. Of mice and not men: differences between mouse and human immunology. J Immunol (2004) 172:2731–8. 10.4049/jimmunol.172.5.2731 14978070

[17] Akkina R. Human immune responses and potential for vaccine assessment in humanized mice. Curr Opin Immunol (2013) 25:403–9. 10.1016/j.coi.2013.03.009 23628166 PMC3894824

[18] Danner R Chaudhari SN Rosenberger J Surls J Richie TL Brumeanu T-D Expression of HLA class II molecules in humanized NOD.Rag1KO.IL2RgcKO mice is critical for development and function of human T and B cells. PLoS One (2011) 6:e19826. 10.1371/journal.pone.0019826 21611197 PMC3096643

[19] Majji S Wijayalath W Shashikumar S Pow-Sang L Villasante E Brumeanu TD Differential effect of HLA class-I versus class-II transgenes on human T and B cell reconstitution and function in NRG mice. Sci Rep (2016) 6:28093. 10.1038/srep28093 27323875 PMC4914985

[20] Wijayalath W Majji S Kleschenko Y Pow-Sang L Brumeanu TD Villasante EF Humanized HLA-DR4 mice fed with the protozoan pathogen of oysters *Perkinsus marinus* (Dermo) do not develop noticeable pathology but elicit systemic immunity. PLoS One (2014) 9:e87435. 10.1371/journal.pone.0087435 24498105 PMC3909113

[21] Allam A Majji S Peachman K Jagodzinski L Kim J Ratto-Kim S TFH cells accumulate in mucosal tissues of humanized-DRAG mice and are highly permissive to HIV-1. Sci Rep (2015) 5:10443. 10.1038/srep10443 26034905 PMC4451806

[22] Mendoza M Ballesteros A Qi Q Sang LP Shashikumar S Casares S Generation and testing anti-influenza human monoclonal antibodies in a new humanized mouse model (DRAGA: HLA-A2. HLA-DR4. Rag1 KO. IL-2Rγc KO. NOD). Hum Vaccin Immunother (2017) 14(2):345–60. 10.1080/21645515.2017.1403703 29135340 PMC5806689

[23] Yi G Xu X Abraham S Petersen S Guo H Ortega N A DNA vaccine protects human immune cells against Zika virus infection in humanized mice. EBioMedicine (2017) 25:87–94. 10.1016/j.ebiom.2017.10.006 29033368 PMC5704055

[24] Majji S Wijayalath W Shashikumar S Brumean TD Casares SA. Humanized DRAGA mice immunized with *Plasmodium falciparum* sporozoites and chloroquine elicit protective pre-erythrocytic immunity. Malar J (2018) 17:114. 10.1186/s12936-018-2264-y 29540197 PMC5853061

[25] Kim J Peachman KK Jobe O Morrison EB Allam A Jagodzinski L Tracking human immunodeficiency virus-1 infection in the humanized DRAG mouse model. Front Immunol (2017) 8:1405. 10.3389/fimmu.2017.01405 29163484 PMC5663722

[26] Chan TC Jiang J Temenak JJ Richards AL. Development of a rapid method for determining the infectious dose (ID)50 of *Orientia tsutsugamushi* in a scrub typhus mouse model for the evaluation of vaccine candidates. Vaccine (2003) 21:4550–4. 10.1016/S0264-410X(03)00505-X 14575767

[27] Chao C-C Belinskaya T Zhang Z Ching W-M. Development of recombinase polymerase amplification assays for detection of *Orientia tsutsugamushi* or *Rickettsia typhi*. PLoS Negl Trop Dis (2015) 9:e0003884. 10.1371/journal.pntd.0003884 26161793 PMC4498641

[28] Jiang J Chan T-C Temenak JJ Dasch GA Ching W-M Richards AL. Development of a quantitative real-time polymerase chain reaction assay specific for *Orientia tsutsugamushi*. Am J Trop Med Hyg (2004) 70:351–6. 10.4269/ajtmh.2004.70.351 15100446

[29] Keller CA Hauptmann M Kolbaum J Gharaibeh M Neumann M Glatzel M Dissemination of *Orientia tsutsugamushi* and inflammatory responses in a murine model of scrub typhus. PLoS Negl Trop Dis (2014) 8:e3064. 10.1371/journal.pntd.0003064 25122501 PMC4133189

[30] Kamala T. Hock immunization: a humane alternative to mouse footpad injections. J Immunol Methods (2007) 328:204–14. 10.1016/j.jim.2007.08.004 17804011 PMC2464360

[31] Turner MD Nedjai B Hurst T Pennington DJ. Cytokines and chemokines: at the crossroads of cell signalling and inflammatory disease. Biochim Biophys Acta (2014) 1843:2563–82. 10.1016/j.bbamcr.2014.05.014 24892271

[32] Robinson DM Huxsoll DL. Protection against scrub typhus infection engendered by the passive transfer of immune sera. Southeast Asian J Trop Med Public Health (1975) 6:477–82. 818716

[33] Palmer BA Hetrick FM Jerrells TJ. Production of gamma interferon in mice immune to *Rickettsia tsutsugamushi*. Infect Immun (1984) 43:59–65. 6317573 10.1128/iai.43.1.59-65.1984 PMC263388

[34] Palmer BA Hetrick FM Jerrells TR. Gamma interferon production in response to homologous and heterologous strain antigens in mice chronically infected with *Rickettsia tsutsugamushi*. Infect Immun (1984) 46:237–44. 6434427 10.1128/iai.46.1.237-244.1984 PMC261462

[35] Eisenberg GH Osterman JV. Gamma-irradiated scrub typhus immunogens: development and duration of immunity. Infect Immun (1978) 22:80–6. 103828 10.1128/iai.22.1.80-86.1978 PMC422119

[36] Jerrells TR Palmer BA Osterman JV. Gamma-irradiated scrub typhus immunogens: development of cell-mediated immunity after vaccination of inbred mice. Infect Immun (1983) 39:262–9. 6185433 10.1128/iai.39.1.262-269.1983 PMC347935

[37] Iwasaki H Mizoguchi J Takada N Tai K Ikegaya S Ueda T. Correlation between the concentrations of tumor necrosis factor-alpha and the severity of disease in patients infected with *Orientia tsutsugamushi*. Int J Infect Dis (2010) 14:e328–33. 10.1016/j.ijid.2009.06.002 19699129

[38] Chung DR Lee YS Lee SS. Kinetics of inflammatory cytokines in patients with scrub typhus receiving doxycycline treatment. J Infect (2008) 56:44–50. 10.1016/j.jinf.2007.09.009 17976731

[39] Del Prete G De Carli M Almerigogna F Giudizi MG Biagiotti R Romagnani S. Human IL-10 is produced by both type 1 helper (Th1) and type 2 helper (Th2) T cell clones and inhibits their antigen-specific proliferation and cytokine production. J Immunol (1993) 150:353–60. 8419468

[40] Astrup E Janardhanan J Otterdal K Ueland T Prakash JAJ Lekva T Cytokine network in scrub typhus: high levels of interleukin-8 are associated with disease severity and mortality. PLoS Negl Trop Dis (2014) 8:e2648. 10.1371/journal.pntd.0002648 24516677 PMC3916254

[41] Soong L Wang H Shelite TR Liang Y Mendell NL Sun J Strong type 1, but impaired type 2, immune responses contribute to *Orientia tsutsugamushi*-induced pathology in mice. PLoS Negl Trop Dis (2014) 8:e3191. 10.1371/journal.pntd.0003191 25254971 PMC4177881

[42] Zlotnik A Yoshie O Chemokines: a new classification system and their role in immunity. Immunity (2000) 12:121–7. 10.1016/S1074-7613(00)80165-X 10714678

[43] Cho B-A Ko Y Kim Y-S Kim S Choi M-S Kim I-S Phenotypic characterization of peripheral T cells and their dynamics in scrub typhus patients. PLoS Negl Trop Dis (2012) 6:e1789. 10.1371/journal.pntd.0001789 22905277 PMC3419201

[44] Boer MC Joosten SA Ottenhoff THM. Regulatory T-cells at the interface between human host and pathogens in infectious diseases and vaccination. Front Immunol (2015) 6:217. 10.3389/fimmu.2015.00217 26029205 PMC4426762

[45] Hauptmann M Kolbaum J Lilla S Wozniak D Gharaibeh M Fleischer B Protective and pathogenic roles of CD8+ T lymphocytes in murine *Orientia tsutsugamushi* infection. PLoS Negl Trop Dis (2016) 10:e0004991. 10.1371/journal.pntd.0004991 27606708 PMC5015871

[46] Xu G Mendell NL Liang Y Shelite TR Goez-Rivillas Y Soong L CD8+ T cells provide immune protection against murine disseminated endotheliotropic *Orientia tsutsugamushi* infection. PLoS Negl Trop Dis (2017) 11:e0005763. 10.1371/journal.pntd.0005763 28723951 PMC5536391

[47] Heuts F Gavier-Widén D Carow B Juarez J Wigzell H Rottenberg ME. CD4+ cell-dependent granuloma formation in humanized mice infected with mycobacteria. Proc Natl Acad Sci U S A (2013) 110:6482–7. 10.1073/pnas.1219985110 23559373 PMC3631626

[48] Brehm MA Wiles MV Greiner DL Shultz LD. Generation of improved humanized mouse models for human infectious diseases. J Immunol Methods (2014) 410:3–17. 10.1016/j.jim.2014.02.011 24607601 PMC4155027

