# Supplementary figures and images for: Dissemination of Orientia tsutsugamushi, a Causative Agent of Scrub Typhus, and Immunological Responses in the Humanized DRAGA Mouse
Source: Front Immunol. 2018 Apr 30;9:816. doi: 10.3389/fimmu.2018.00816 (PMC5936984; doi:10.3389/fimmu.2018.00816)

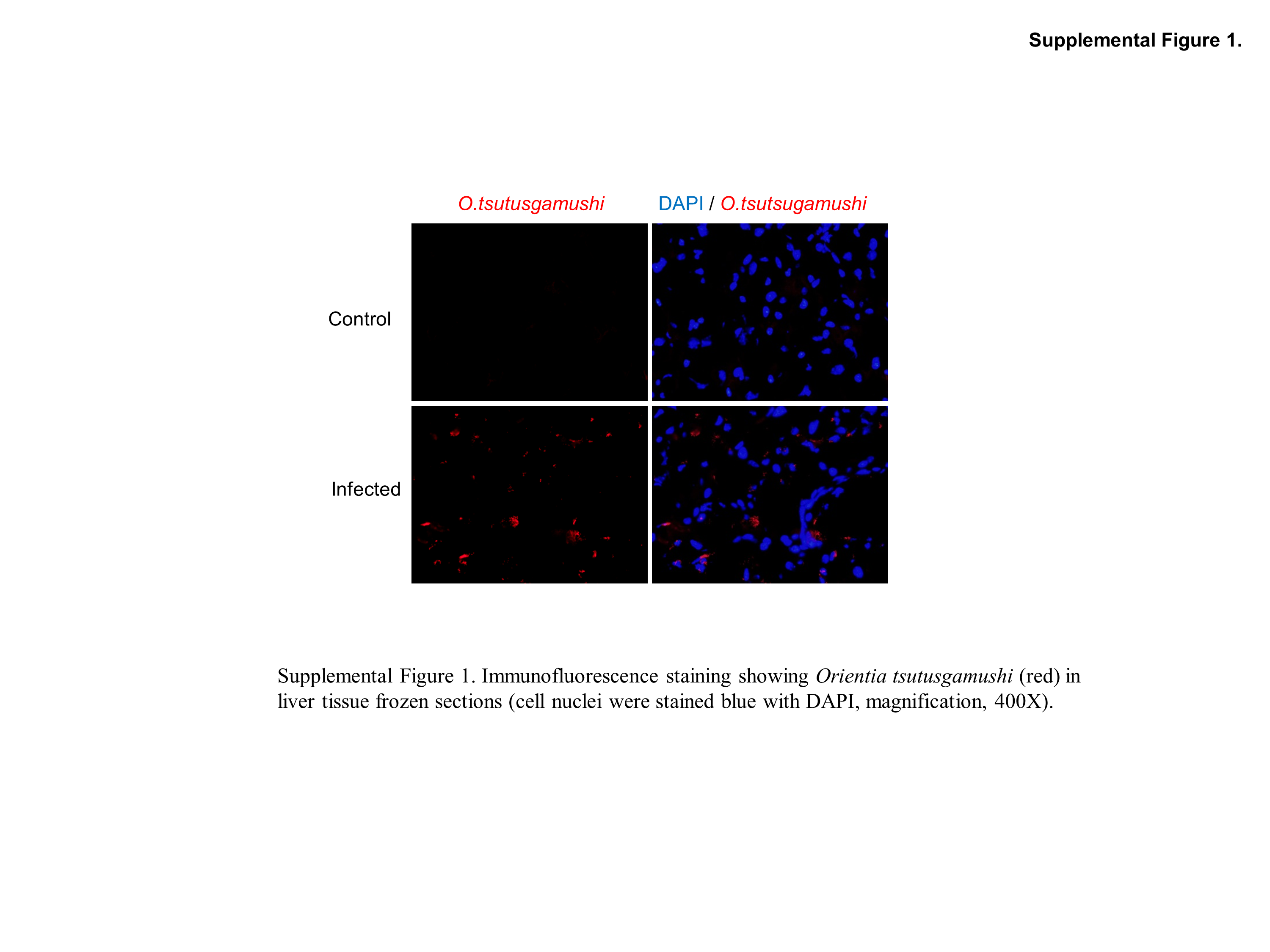

Supplement: Supplementary file 1 [file image_1.tif]

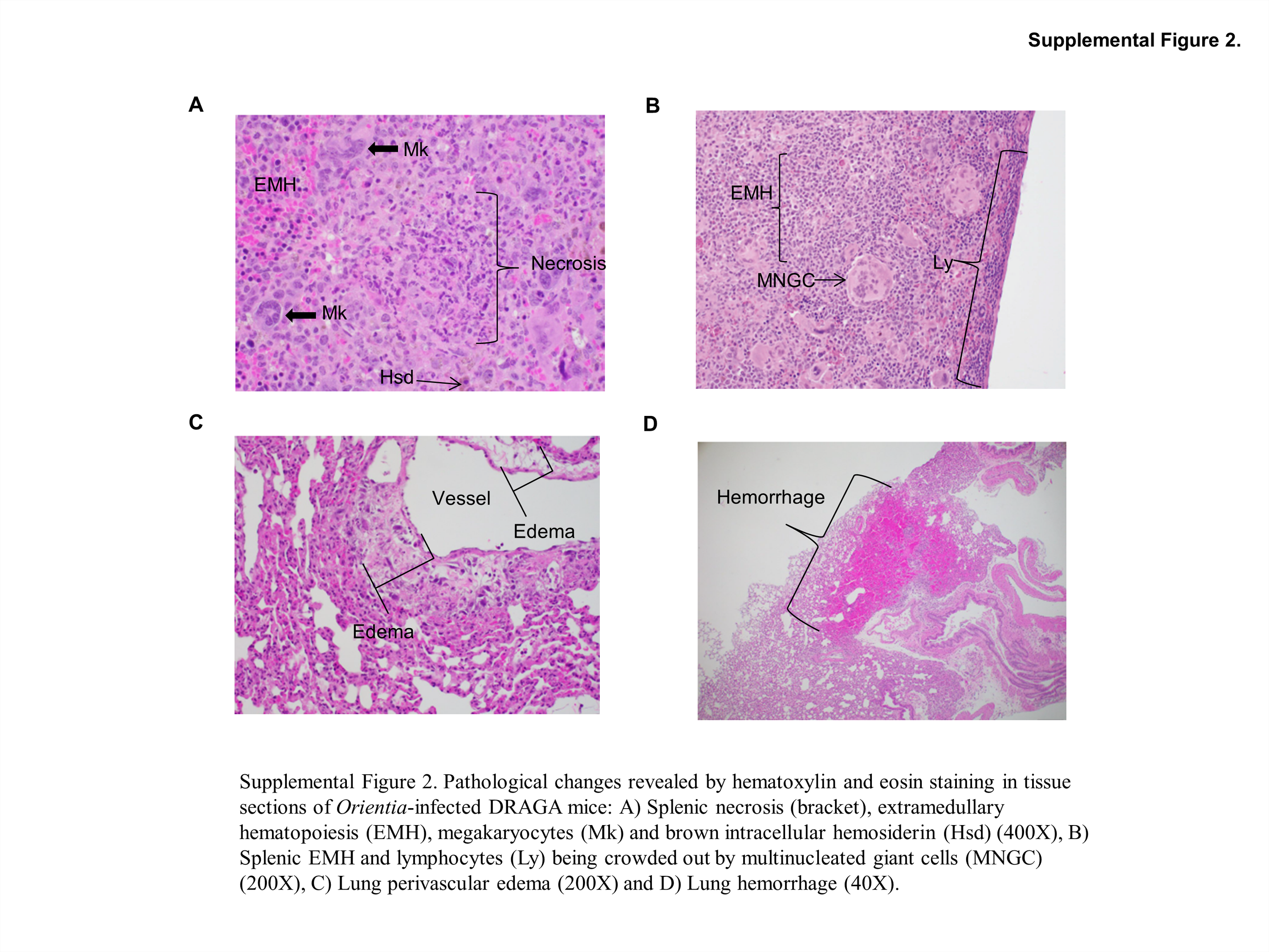

Supplement: Supplementary file 2 [file image_2.tif]

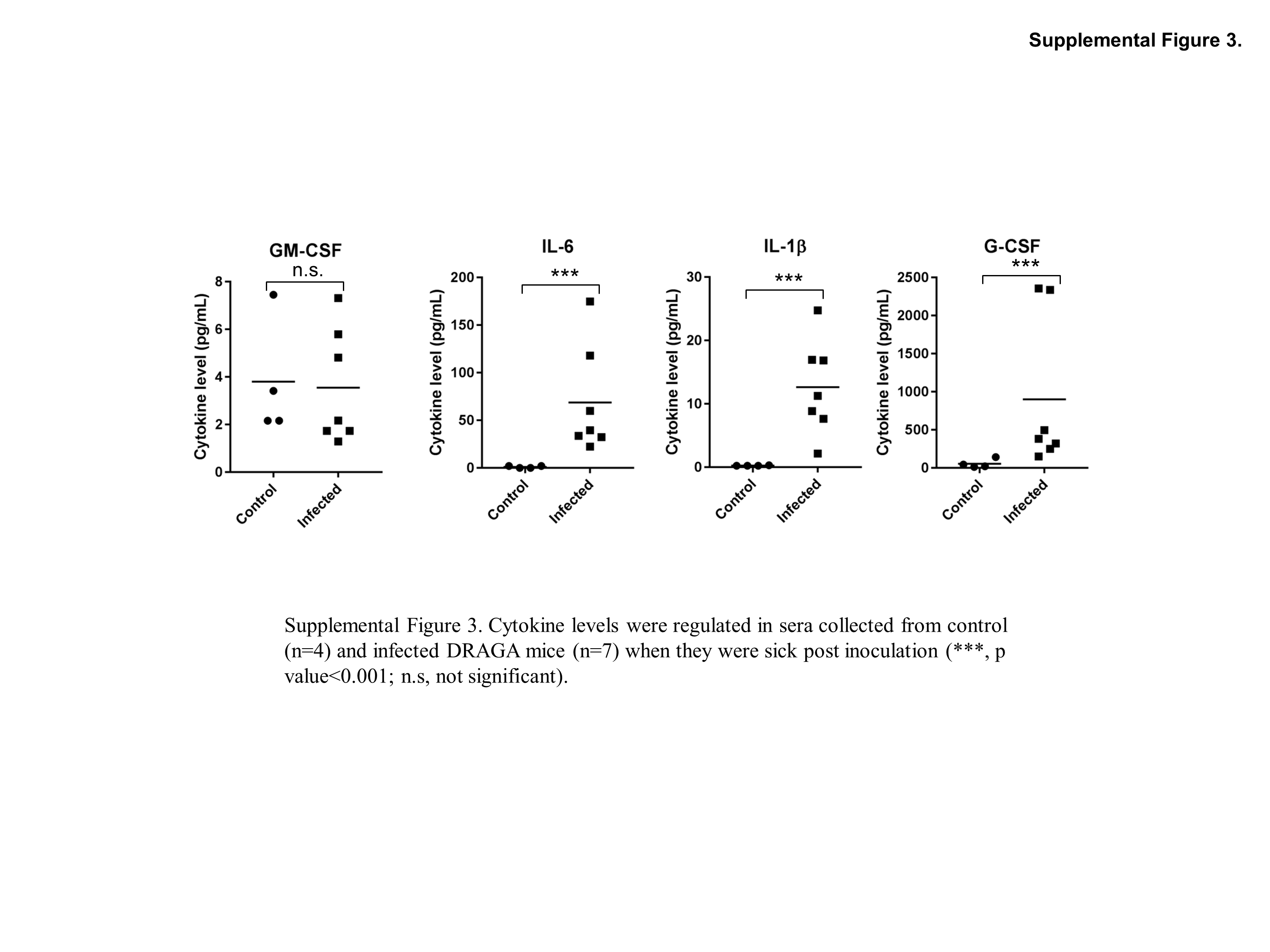

Supplement: Supplementary file 3 [file image_3.tif]

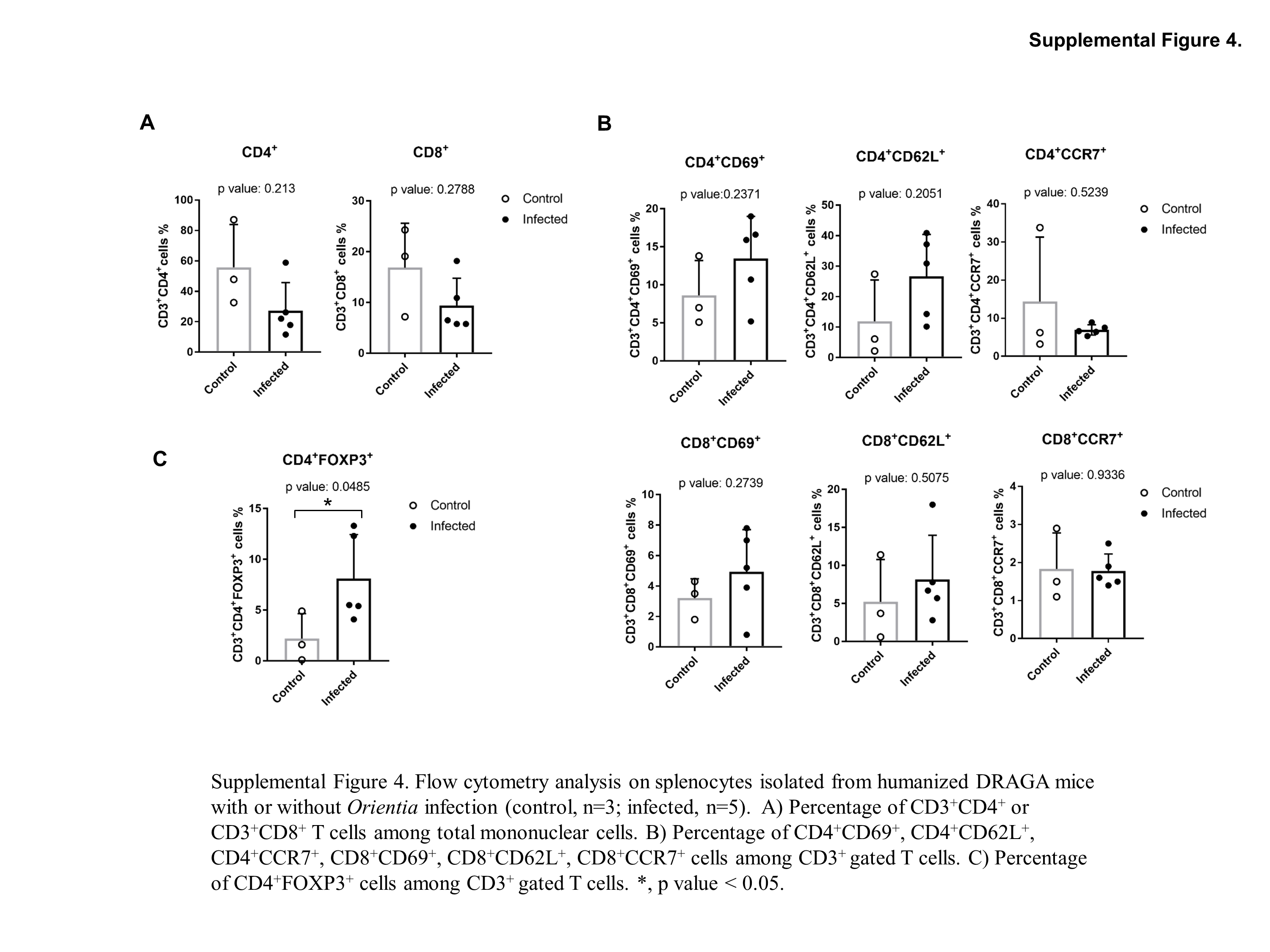

Supplement: Supplementary file 4 [file image_4.tif]

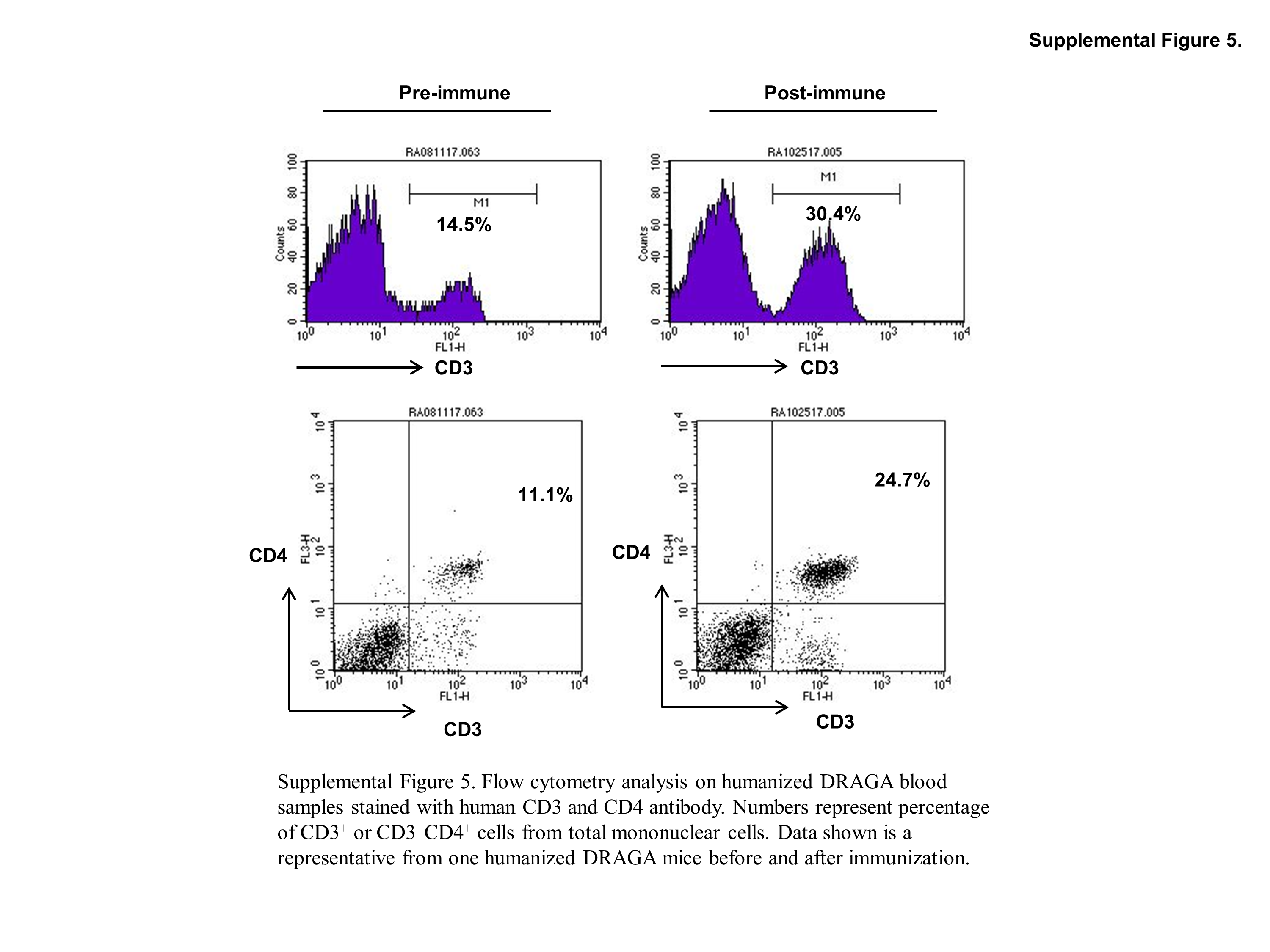

Supplement: Supplementary file 5 [file image_5.tif]
